# What CVD risk factors predict self-perceived risk of having a myocardial infarction? A cross-sectional study

**DOI:** 10.1016/j.ijcrp.2022.200125

**Published:** 2022-01-13

**Authors:** Åsa Grauman, Liisa Byberg, Jorien Veldwijk, Stefan James

**Affiliations:** a Centre for Research Ethics & Bioethics, Uppsala University, Uppsala, Sweden; b Department of Surgical Sciences, Medical Epidemiology, Uppsala University, Uppsala, Sweden; c Erasmus School of Health Policy & Management, Erasmus University, Rotterdam, the Netherlands; d Erasmus Choice Modelling Centre, Erasmus University, Rotterdam, the Netherlands; e Department of Medical Sciences, Cardiology, Uppsala University, Uppsala, Sweden

**Keywords:** Risk perception, Relative risk, Absolute risk, Myocardial infarction, CVD risk Factors, General population

## Abstract

**Background:**

This study aims to identify predictors of self-perceived risk of myocardial infarction (MI).

**Methods:**

Among 564 men and women (50–65 years; randomly selected from the Swedish population), we assessed risk perception as relative self-perceived risk compared to others (lower, same, higher) and percentage ten-year absolute risk. Predictors (added blockwise) were identified using multinomial or linear regression, providing odds ratios (ORs) or β coefficients with their 95% confidence intervals (CI).

**Results:**

The mean of self-perceived 10-year MI risk was 12%. Lower BMI (AOR 0.57, 95% CI: 0.44–0.75), low stress (AOR 2.51, 95% CI: 1.39–4.52), high level of physical activity (AOR 1.66, 95% CI:1.01–2.74), hypertension (AOR 0.42, 95% CI: 0.23–0.76), family history (AOR 0.38, 95% CI: 0.21–0.69), and poor general health (AOR 0.41, 95% CI: 0.19–0.89) predicted if respondents perceived their MI risk as lower. Poor general health (AOR 1.94, 95% CI: 1.01–3.73), family history (AOR 2.72, 95% CI: 1.57–4.72), and high cholesterol (AOR 2.45, 95% CI: 1.18–5.09) predicted if respondents perceived their MI risk as higher. Low level of self-perceived CVD knowledge and low numeracy predicted if respondents perceived their MI risk as the same as others. High cholesterol (B 6.85, 95% CI: 2.47–11.32) and poor general health (B 8.75, 95% CI: 4.58–13.00) predicted a higher percentage of perceived ten-year risk.

**Conclusion:**

General health was a common predictor of self-perceived MI risk. Lifestyle factors (BMI, physical activity) and stress dominated the predictors for perceiving MI risk as lower than others, while high cholesterol predicted perception of high risk.

## 1 Introduction

Cardiovascular diseases (CVDs) are the number one cause of death worldwide [1]. The majority of the factors affecting CVDs are modifiable, e.g., smoking, hypertension, diabetes, high cholesterol level, obesity, negative psychosocial factors, diet, and exercise [2]. To facilitate risk assessment in clinical practice, physicians are recommended to use risk prediction models that include several established risk factors [3]. To be successful in preventing and managing CVD risk, it is crucial to mobilize the individual, since it is the individual, to a large extent, who can manage the risk in his/her everyday life. Perceived risk is one of the main components that explain health behavior [4]. Individuals often have an inaccurate risk perception [5], which is problematic since patients that accurately perceive their risk as high reported higher compliance with secondary prevention interventions [6]. Furthermore, high perceived risk has shown to predict positive change in risk factors in a community intervention [7]. Therefore, knowledge of which risk factors predict risk perception is important since it can guide risk communication in clinical practice. Similarly, it is useful information to have when designing health communication campaigns for the public.

A Brazilian study of people aged >40 years found that CVD risk perception was associated with BMI, blood pressure, and diabetes [8]. A study of the U.S. general population, aged 18–65, found high stress, general health, and family history of CVD to be associated with high CVD risk perception [9]. Family history, high cholesterol, lack of physical activity were also associated with CVD risk perception in a study from the Netherlands, where the study population comprised individuals, aged 57–79, identified as high-risk for diabetes and CVD [10].

The format in which risks are presented plays a crucial role in how individuals perceive risks, their emotional response to the risks, their intention to modify behavior, and their decisions about accepting medical treatment [11,12]. It is therefore reasonable to expect that different formats influence how individuals express their self-perceived risk. Most previous studies have used categorical (verbal) measurements of CVD risk perception. Others have asked the participants to rate their perceived risk in percentages, using a numerical vas scale with the range 0–100 [13] which is comparable to the risk numbers presented in risk estimation models such as HeartScore (Systematic Coronary Risk Estimation). However, individuals, in general, have a very hard time dealing with probabilities and numerical information [11,14,15]. Furthermore, only using a numerical measurement makes it difficult to know what the participant thinks of the number he or she stated, in terms of low, moderate, and high. Previous studies have been inconclusive about which factors predict an individual's risk perception, and furthermore used either numerical or categorical measurements.

Therefore, the aim of this study is to identify what individual factors predict the participants’ self-perceived risk of experiencing a myocardial infarction (MI) among the Swedish population, aged 50–65, using both a categorical and a numerical measurement of self-perceived risk.

## 2 Methods

### 2.1 Data collection & study population

This was a cross-sectional study. The data collection has been described in detail previously and is based on the Swedish CArdioPulmonaryBioImage Study (SCAPIS) [16]. This study sample was chosen for convenience reasons. The SCAPIS participants (age 50–64 years) were randomly selected from the Swedish population, with the purpose of creating a cohort for the study of chronic obstructive pulmonary disease (COPD) and cardiovascular disease (CVD). Exclusion criterion was inability to understand spoken and written Swedish for informed consent. The data for the present study were collected among 615 consecutive SCAPIS participants from February to March 2017, using a web-based questionnaire [17]. All data was self-reported with the exception for weight and height used for the calculation of Body Mass Index (BMI) that were measured in the SCAPIS health examinations. The study was approved by the Uppsala Regional Ethical Review Board (2016/256).

### 2.2 Measurements

Self-perceived risk was assessed using two different measures: numerical and categorical. The numerical measure was assessed with the question “What percentage best describes your risk of having a heart attack within the next ten years?” Respondents were asked to choose the percentage from a set of options (0%, 2%, 5%; then the options were even tens: 10%, 20%, 30%, etc. continuing to 100%). The categorical risk perception measure was assessed with a 7-point Likert scale using the question “Compared to other people of the same age and sex as you, how do you perceive your risk of having a heart attack in the next ten years?” It was collapsed into three categories: lower than others (1–3 points including much lower risk, moderately lower risk, slightly lower risk), same as others (4 points including neither lower nor higher risk), and higher than others (5–7 points including slightly higher risk, moderately higher, much higher).

Experience of CVD (angina, atrial fibrillation, heart failure, valvular, bypass or angioplasty, atrial occlusive disease, stroke, myocardial infarction) diabetes, hypertension, or high cholesterol was defined as being diagnosed and/or treated. Family history of MI included siblings and/or parents. Chest pain was assessed using the question “Do you get a tingling feeling or pain in the chest when you go up hills or stairs, or when you walk fast on flat ground?” Shortness of breath was assessed with the question “Do you suffer from shortness of breath when you are in a hurry and walk on level ground or when you go up a small slope?”

Smoking was dichotomized into yes (daily or occasional smoking) and no (stopped smoking or never smoked). Self-perceived stress was collapsed into low level of stress (‘never experienced stress’, ‘experienced some periods of stress’, and ‘experienced a period of stress the lasted five years’) and high level of stress (‘constant stress the last year’ and ‘constant stress during the last five years’). Physical activity was measured using the question: “How often do you exercise?” The five-point item was dichotomized into low levels of physical activity (‘never’, ‘seldom’, ‘1–2 times/week’) and high levels of physical activity (’2 times/week or more’). General health was assessed through the question “In general, would you say your health is: excellent, very good, good, somewhat good, or poor?” and dichotomized into poor (‘somewhat good’ and ‘poor’) and good (‘good’, ‘very good’, and ‘excellent’).

Health literacy (HL) was measured using the validated Swedish version of The Communicative and Critical Health Literacy scale (S–C & C HL scale). It has five-items and a five-point Likert scale [18]. Participants who only answered ‘strongly agree’ or ‘agree’ were categorized as having sufficient HL; those who answered ‘strongly agree,’ ‘agree,’ or ‘partially agree’ were categorized as having problematic HL; and those who answered ‘strongly disagree’ or ‘disagree’ on any item were categorized as having inadequate HL. Self-perceived knowledge of factors that can increase or decrease the risk of experiencing a myocardial infarction was dichotomized into a little (very little, fairly little, and neither a lot nor a little) and a lot (fairly much and very much). Numeracy was assessed by using the Short 3-item Version of Subjective Numeracy Scale, a three-item, five-point Likert scale, from which a mean summary score was calculated [19].

### 2.3 Statistical analysis

Descriptive statistics are presented with mean and standard deviation for continuous variables and as frequencies for categorical variables. Associations of sociodemographic factors, health literacy, CVD knowledge, and CVD risk factors were tested in univariate analyses using T-tests and Pearson's correlations for the linear risk perception variable, and the Chi square test and one-way ANOVA for the categorical risk perception variable. Associations were established at a 95% CI level and p < 0.05 was considered statistically significant. Missing data was handled by partial deletion of participants missing data on categorical risk perception variable (n = 51). Full analysis was performed on the reduced data set (n = 564). Collinearity diagnoses showed that the variance inflation factor ranged between 1.07 and 1.40, indicating that multicollinearity was not an issue.

Multinomial regression was used to identify predictors of relative categorical risk perception. Outcomes were lower risk and higher risk (same risk was reference category). Multiple linear regression was used to identify predictors of perception of ten-year total MI risk. Variables were added in blocks to a basic model, including age and sex. The contribution of the blocks to the overall model was evaluated using Log likelihood ratio tests for multinomial regression and the F-test for multiple linear regression. The first block included lifestyle- and psychosocial factors (stress, smoking, BMI, and physical activity). The second block included clinical risk factors (CVD, diabetes, high cholesterol, hypertension, and family history of MI). The third block included CVD symptoms and self-perceived health (chest pain, shortness of breath, and general health). The fourth block included educational level, health literacy, numeracy, and self-perceived knowledge of CVD risk factors. Standardized odds ratios (ORs) and β coefficients are given for continuous variables (per SD increase). All analyses were performed using SPSS 27.

## 3 Results

Characteristics of the respondents are presented in Table 1. About half of the respondents had a university degree, 8% were smokers, and about half never or seldom exercised.

**Table 1:** Descriptive statistics; characteristics of the respondents and univariate associations with risk perception. Associations with numerical risk perception are expressed as Pearson's correlation coefficient for continuous variables and mean (SD) in each level for categorical variables and p-values. n = 564.

Variables (n)	Descriptive statistics of respondents^a^	Risk perception numerical^b^		Risk perception categorical^a^			
			p-value^c^	Lower risk	Neither lower nor higher risk	Higher risk	p-value^d^
**Age (**years), mean (SD) (564)	57.8 (4.4)	0.10	0.03	57.56 (4.4)	58.3 (4.5)	57.5 (4.3)	0.11
**Sex (564)**			0.43				0.11
Male	264 (46.8)	12.50 (15.8)		111 (49.1)	89 (42.4)	64 (52.0)	
Female	300 (53.2)	11.38 (15.5)		115 (50.9)	126 (58.6)	59 (48.0)	
**Education (564)**			0.06				0.01
Primary or secondary school	285 (50.5)	13.38 (17.1)		102 (45.1)	127 (59.1)	56 (45.5)	
University level	279 (49.5)	10.53 (14.0)		124 (54.9)	88 (40.9)	67 (54.5)	
**Health literacy (564)**			0.00				0.00
Sufficient	346 (61.3)	10.32 (14.4)		157 (69.5)	116 (54.0)	73 (59.3)	
Problematic or Inadequate	218 (38.7)	14.73 (17.3)		69 (30.5)	99 (46.0)	50 (40.7)	
**Numeracy, mean (SD) (564)**	3.8 (0.9)	−0.039	0.39	3.9 (0.8)	3.6 (0.8)	4.0 (0.8)	<0.00
**Self-perceived CVD risk factor knowledge (563)**			0.16				<0.00
A little	210 (37.3)	13.20 (16.5)		66 (29.3)	109 (50.7)	35 (28.5)	
A lot	353 (62.7)	11.15 (15.1)		159 (70.7)	106 (49.3)	88 (71.5)	
**Smoker (554)**			0.03				0.24
No	509 (91.9)	11.57 (15.1)		210 (94.2)	189 (90.9)	110 (89.4)	
Yes	45 (8.1)	17.31 (20.4)		13 (5.8)	19 (9.1)	13 (10.6)	
**Physical activity (560)**			0.01				<0.00
Never, seldom, 1–2 times a week	388 (68.8)	13.10 (16.1)		130 (57.5)	164 (77.4)	94 (77.0)	
2 times a week or more	172 (30.5)	9.38 (14.6)		96 (42.5)	48 (22.6)	28 (23.0)	
**Stress (561)**			0.05				<0.00
Never or periods of stress	447 (79.7)	11.24 (15.4)		197 (87.6)	162 (75.7)	88 (72.1)	
Constant stress	114 (20.3)	14.87 (16.5)		28 (12.4)	52 (24.3)	34 (27.9)	
**BMI (kg/m**^**2**^**),** mean (SD) (564)	27.1 (4.1)	0.22	<0.00	25.67 (3.7)	27.71 (4.0)	28.5 (4.4)	<0.00
**General health (564)**			<0.00				<0.00
Poor, Somewhat good	101 (18.4)	21.54 (20.3)		213 (94.2)	166 (77.2)	81 (65.9)	
Good, Very good, Excellent	460 (81.5)	10.03 (13.8)		13 (5.8)	49 (22.8)	42 (34.1)	
**Diabetes (561)**			0.02				<0.00
No	541 (96.4)	11.60 (15.5)		225 (100)	207 (96.7)	109 (89.3)	
Yes	20 (3.6)	21.60 (18.0)		0 (0)	7 (3.3)	13 (10.7)	
**Hypertension (561)**			0.01				<0.00
No	433 (77.2)	10.92 (15.0)		199 (88.4)	152 (71.0)	82 (67.2)	
Yes	128 (22.8)	15.50 (17.5)		26 (11.6)	62 (29.0)	40 (32.8)	
**High cholesterol (561)**			<0.00				<0.00
No	495 (88.2)	10.80 (14.9)		214 (95.1)	191 (89.3)	90 (73.8)	
Yes	66 (11.8)	19.77 (18.80)		11 (4.9)	23 (10.7)	32 (26.2)	
**CVD**^e^**(561)**			0.05				0.01
No	528 (94.8)	11.56 (15.42)		215 (95.69	205 (95.8)	108 (88.5)	
Myocardial infarction	7 (1.2)	17.4 (18.7)		10 (4.4)	9 (4.2)	14 (11.5)	
Stroke	8 (1.4)						
Atrial fibrillation	6 (1.1)						
Other CVD	12 (2.1)						
Total	33 (5.9)						
**Family history of MI (549)**			<0.00				<0.00
No	410 (74.7)	10.63 (14.4)		189 (85.5)	156 (75.7)	65 (53.3)	
Yes	139 (25.3)	15.72 (18.4)		32 (14.5)	50 (24.3)	57 (46.7)	
**Shortness of breath**^f^**(560)**			<0.00				0.02
No	522 (93.2)	11.12 (15.04)		218 (96.5)	196 (92.5)	108 (88.5)	
Yes	38 (6.8)	24.00 (20.12)		8 (3.5)	16 (7.5)	14 (11.5)	
**Chest pain**^g^**(560)**			<0.00				<0.00
No	546 (97.5)	11.55 (15.3)		225 (99.6)	210 (99.1)	11 (9.0)	
Yes	14 (2.5)	25.58 (20.4)		1 (0.4)	2 (0.9)		

a numbers are N (%) unless otherwise stated.

b Associations are expressed as Pearson's correlation coefficient for continuous variables and mean (SD) in each level for categorical variables.

c p-value from Pearson's correlation (continuous variables) or T-test (categorical variables).

d p-value from ANOVA (continuous variables) or Chi^2^ test (categorical variables).

e angina (n = 3), heart failure (n = 3), valvular (n = 4), bypass or angioplasty (n = 1), arterial occlusive disease (n = 1).

f When in a hurry or go up a small slope.

g When you go up hills or stairs, or when you walk fast on flat ground.

### 3.1 Distribution of MI risk perception

**Fig. 1:**
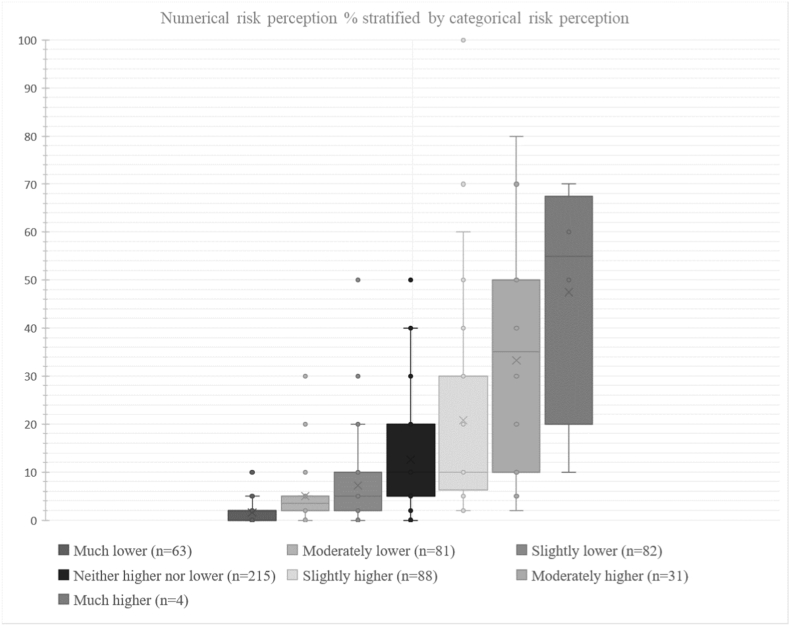
Distribution of self-perceived risk. Perceived risk in percentages (n = 483) stratified on categorical self-perceived risk (n = 564).

Most respondents perceived their MI risk as either lower (40.1%) or the same as others (38.1%). The mean level of self-perceived numerical risk was 12.0% (SD 15.7). The two measurements were correlated; the mean of the numerical risk perception increased for every step on the categorical risk perception scale (Fig. 1).

### 3.2 Categorical risk perception

Univariate analyses revealed that a low level of physical activity, high level of stress, higher BMI, diabetes, hypertension, high cholesterol, CVD, family history of MI, worse general health, shortness of breath, and chest pain were associated with high perceived risk. Individuals with lower levels of education, knowledge of CVD risk factors, health literacy, and numeracy were more likely to perceive their risk as same as others, compared to lower or higher MI risk. No associations were found for sex, age, and smoking (Table 1).

The multinomial regression analysis showed that all blocks contributed to predicting risk perception. Together, the blocks of variables explained 42.9% of the variance in risk perception. Low perceived stress, low BMI, regular physical activity, lack of hypertension, and being male predicted perceiving MI risk lower than others. Lack of family history and good general health predicted lower risk perception and vice versa. Low numeracy and self-perceived knowledge predicted perceiving MI risk same as others. High cholesterol predicted perceiving MI risk higher than others (Table 2).

**Table 2:** Odds ratio with 95% confidence intervals of predictors for perceiving MI risk lower or higher than others. Standardized Odds ratio for continuous variables. Results from a multinomial regression analysis using same risk as reference category.

Predictor	Model 1: age and sex		Model 2: Model 1 + Lifestyle and psychosocial factors		Model 3: Model 2 + Clinical risk factors		Model 4: Model 3 + Symptoms and general health		Model 5: Model 4 + Education, health literacy, CVD knowledge, numeracy	
Nagelkerke R^2^	1.7%		16.1%		34.5%		38.5%		42.9%	
**Df**	2		12		22		28		36	
**p-value**^a^	n.a.		<0.05		<0.05		<0.05		<0.05	
	*Lower*	*Higher*	*Lower*	*Higher*	*Lower*	*Higher*	*Lower*	*Higher*	*Lower*	*Higher*
*Age (years)*^b^	0.84 (0.70, 1.02)	0.82 (0.66, 1.03)	0.80 (0.63, 0.98	0.81 (0.64, 1.02)	0.83 (0.67, 1.03)	0.74 (0.58, 0.96)	0.81 (0.65, 1.01)	0.77 (0.59, 1.00)	0.82 (0.67, 1.05)	0.80 (0.61, 1.04)
*Sex (male)*	1.36 (0.93, 1.99)	1.53 (0.99, 2.39)	1.56 (1.03, 2.37)	1.62 (1.02, 2.57)	1.90 (1.22, 2.95)	1.43 (0.86, 2.36)	2.00 (1.27, 3.15)	1.58 (0.93, 2.68)	1.94 (1.19, 3.15)	1.53 (0.87, 2.69)
Physical activity (two times a week of more)			1.88 (1.20, 2.93)	1.05 (0.60, 1.82)	1.98 (1.23, 3.18)	1.02 (0.56, 1.85)	1.81 (1.11, 2.95)	1.12 (0.60, 2.10)	1.66 (1.01, 2.74)	0.95 (0.50, 1.81)
Stress (low)			2.13 (1.25, 3.63)	0.84 (0.50, 1.43)	2.62 (1.50, 4.60)	0.85 (0.48, 1.50)	2.52 (1.41, 4.49)	1.06 (0.58, 1.93)	2.51 (1.39, 4.52)	1.12 (0.60, 2.08)
BMI (kg/m^2^)^b^			0.57 (0.46, 0.72)	1.13 (0.90, 1.41)	0.58 (0.46, 0.74)	1.15 (0.90, 1.47)	0.58 (0.45, 0.75)	1.09 (0.84, 1.42)	0.57 (0.44, 0.75)	1.10 (0.84, 1.44)
Smoke			0.59 (0.27, 1.32)	1.17 (0.55, 2.50)	0.60 (0.26, 1.36)	1.32 (0.59, 2.93)	0.66 (0.28, 1.56)	1.22 (0.55, 2.77)	0.82 (0.34, 1.99)	1.52 (0.64, 3.62)
Hypertension					0.38 (0.22, 0.70)	0.76 (0.42, 1.37)	0.42 (0.23, 0.75)	0.63 (0.34, 1.17)	0.42 (0.23, 0.76)	0.71 (0.38, 1.34)
High cholesterol					0.44 (0.18, 1.03)	2.61 (1.31, 5.19)	0.45 (0.19, 1.08)	2.59 (1.28, 5.22)	0.38 (0.16, 0.94)	2.45 (1.18, 5.09)
Diabetes^c^					NA	2.54 (0.86, 7.47)	NA	2.72 (0.91, 8.10)	NA	2.52 (0.81, 7.83)
CVD					1.88 (0.63, 5.68)	2.97 (1.10, 8.05)	1.64 (0.52, 5.18)	2.34 (0.82, 6.69)	1.68 (0.53, 5.35)	2.26 (0.78, 6.53)
Family history of MI					0.42 (0.24, 0.73)	2.73 (1.62, 4.57)	0.42 (0.24, 0.74)	2.80 (1.65, 4.76)	0.38 (0.21, 0.69)	2.72 (1.57, 4.72)
General health (poor, somewhat good)							0.38 (0.18, 0.81)	1.77 (0.94, 3.34)	0.41 (0.19, 0.89)	1.94 (1.01, 3.73)
Shortness of breath							2.26 (0.75, 6.79)	1.05 (0.37, 2.96)	2.37 (0.75, 7.52)	1.37 (0.48, 3.95)
Chest pain							0.56 (0.04, 7.57)	4.41 (0.81, 24.16)	0.64 (0.05, 8.05)	4.38 (0.75, 25.39)
Education (university)									0.88 (0.54, 1.42)	1.53 (0.85, 2.74)
Numeracy^b^									1.31 (1.01, 1.70)	1.42 (1.03, 1.94)
Knowledge of CVD risk factors (high)									2.18 (1.35, 3.51)	2.02 (1.15, 3.55)
Health literacy (high)									1.23 (0.74, 2.04)	0.88 (0.49, 1.58)

n.a. Not available.

a p-value from Log-likelihood tests testing whether the new added block of predictors in each model contributes to the full model.

b Estimates are per SD increase.

c No participant with diabetes perceived their MI risk as lower than others.

### 3.3 Numerical risk perception

Univariate analysis showed that higher age, lower health literacy level, higher BMI, smoking, lower level of exercise, diabetes, hypertension, high cholesterol, shortness of breath, chest pain, family history of MI, and worse general health were associated with a higher perceived risk, expressed as a risk percentage. No associations were found for sex, education, knowledge of CVD risk factors, numeracy, stress, and CVD (Table 1).

In the multivariable linear regression, blocks 2, 3, and 4 contributed to predicting numerical risk perception. Together, the variables explained 13.5% of the variance in risk perception. Block 5, which included education, health literacy, numeracy, and self-perceived knowledge, did not contribute and was therefore not included in the model. When adding block 2, stress, smoking, and BMI predicted risk perception. These associations remained stable when block 3 was added. High cholesterol and family history also predicted risk perception when block 3 was added. After adding block 4, only general health and high cholesterol predicted risk perception (Table 3).

**Table 3:** β coefficients for continuous variables and B for categorical variables, and their 95% confidence intervals from multiple linear regression to evaluate predictors of numerical risk perception. N = 465.

	Model 1: age and sex	Model 2: Model 1 + Lifestyle	Model 3: Model 2 + clinical factors	Model 4: Model 3+ Symptoms, perceived health	Model 5: Education, health literacy, CVD knowledge, numeracy
Adjusted R^2^	0.5%	4.8%	8.9%	13.5%	13.4%
R^2^ change	0.01	0.05	0.05	0.05	0.008
p-value^a^	n.a.	<0.001	<0.001	<0.001	0.43
**CVD risk factors**					
Age (years)^b^	0.09 (−0.01, 0.65)	0.10 (0.04, 0.68)	0.08 (−0.05, 0.59)	0.09 (−0.00, 0.62)	0.08 (−0.04, 0.58)
Sex (male)	1.29 (−1.61, 4.18)	0.92 (−1.96, 3.80)	−0.15 (−3.01, 2.70)	0.31 (−2.50, 3.12)	−0.08 (−3.05, 2.89)
Physical activity (≥2 times a week)		−1.30 (−4.47, 1.86)	−1.18 (−4.29, 1.94)	−0.03 (−3.09, 3.04)	0.36 (−2.74, 3.46)
Stress (low)		−4.22 (−7.86, −0.57)	−4.05 (−7.62, −0.47)	−2.25 (−5.81, 1.31)	−2.34 (−5.91, 1.23)
BMI (kg/m^2^)^b^		0.15 (0.20, 0.92)	0.11 (0.07, 0.80)	0.06 (−0.14, 0.61)	0.06 (−0.16, 0.59)
Smoke		6.12 (0.97, 11.27)	6.30 (1.24, 11.36)	4.95 (−0.02, 9.92)	4.72 (−0.32, 9.77)
Hypertension			1.24 (−2.38, 4.85)	0.28 (−3.29, 3.84)	0.27 (−3.32, 3.87)
High cholesterol			7.03 (2.56, 11.50)	6.85 (2.47, 11.32)	7.51 (3.08, 11.94)
Diabetes			4.29 (−3.92, 12.51)	5.41 (−2.62, 13.44)	4.60 (−3.50, 12.69)
CVD			3.63 (−2.62, 9.86)	1.71 (−4.62, 8.03)	1.80 (−4.54, 8.14)
Family history of MI			3.58 (0.34, 6.83)	2.88 (−0.31, 6.06)	2.69 (−0.52, 5.89)
General health (poor, somewhat good)				8.79 (4.58, 13.00)	8.62 (4.39, 12.84)
Shortness of breath				3.41 (−3.19, 10.02)	3.35 (−3.27, 9.98)
Chest pain				5.00 (−4.47, 14.47)	5.04 (−4.50, 14.58)
Education (university)					0.03 (−2.96, 3.03)
Numeracy^b^					0.53 (−1.31, 2.38)
Knowledge of CVD risk factors (high)					−1.18 (−4.15, 1.78)
Health literacy (high)					−2.70 (−5.83, 0.43)

a p-values from F-test testing whether the new added block of predictors in each model contributes to the full model.

b Estimates are per SD increase.

## 4 Discussion

Most respondents in this study perceived their MI risk as lower (40.1%) or equal to (38.1%) that of others. The mean of perceived 10-year MI risk was 12%. Lifestyle and psychosocial factors (lower BMI, low stress, high level of physical activity), as well as hypertension, predicted if respondents perceived their MI risk as lower. High cholesterol predicted if respondents perceived their MI risk as higher to that of others, and a higher percentage of perceived ten-year risk. Family history predicted categorical risk perception, both lower and higher, and general health was identified as a common predictor for all three outcomes used in this study. These two risk factors have repeatedly been identified as predictors of CVD risk perception in previous studies [9,10,13,16]. It may therefore be appropriate to choose general health as a predictor of CVD risk perception, and furthermore, to adjust for general health when studying the association of other risk factors and risk perception. General health should also be considered in clinical practice; it is a good predictor for cardiovascular health but meanwhile from the patient perspective good self-perceived general health can overshadow the presence of other risk factors making the patient underestimate their CVD risk [16]. This tendency may be explained by the fact that people have reported that if they feel fine they are not worried about their CVD [20].

It is worth noting that smoking and CVD were not identified as predictors of MI risk perception. MI-patients have underestimated their risk of having new MI-events in previous studies [6,21]. One explanation could be that these patients respond well to treatment and therefore do not perceive themselves to be at higher risk than others. It could also be that they perceive CVDs as temporary rather than chronic conditions, meaning that after the acute event has passed, their risk for a new event is back at a normal level. This is supported by the findings of a qualitative study, where MI patients viewed their condition as acute and treatment as curative [22]. However, in this study sample, experience of specific CVDs were rather uncommon, only seven participants had experience of MI, resulting in the decision to collapse the different CVDs into one category. Although, it is possible that different CV conditions have different impact on risk perception, and that association of the experience of MI with risk perception was diluted when combining it with other CV conditions. Regarding smoking, Claassen et al. (2011) also did not find an association between being a smoker and risk perception, despite the fact that the participants believed that smoking was a CVD risk factor [10].

The two risk perception measurements used in this study were strongly correlated with each other; respondents that perceived their risk as higher than others also perceived their risk to be higher in numerical terms. The overall model fit for the linear regression was low; the predictors only explained 13.5% of the variation in the numerical measurement, and the range of answers was quite wide. The fact that neither knowledge of CVD risk factors nor numeracy was associated with the numerical measurement suggests that the responses were rather random and indicate difficulties with answering the question. This assumption was strengthened by the fact that more participants skipped the numerical question about risk, which might be an indication that they found it more difficult to answer this compared to the categorical question. Furthermore, both cardiovascular risk and risk perception are multifactorial [23], and it is likely that relevant predictors were not included in this study, such as mental health, alcohol use, personality traits and social support [24].

Furthermore, the mean self-perceived numerical risk was 12%, which according to HEART SCORE is a very high risk [3]. Therefore, if the average person perceives his or her risk to be 12%, but is presented with a risk that is 5%, he or she may think of it as very low, while a 5% risk is in fact considered high according to SCORE. To address this problem, Lipkus (2007) recommends the use of a common reference point, which involves presenting the mean risk for the population, risk for a person without risk factors as well as the risk of another common event, such as a car accident [12]. No common reference point was included with the numerical measurement in this study, which is a limitation since it could have helped the respondents.

Another interesting finding from the univariate analyses was that respondents with low education, self-reported knowledge of CVD risk factors, heath literacy, and numeracy were more likely to respond that they perceived their CVD risk as “same as others”, rather than “lower” or “higher” risk than others. This association remained stable for numeracy and knowledge in the multinomial regression. Sturgis et al. (2014) found that respondents who chose the middle, more neutral, alternative in Likert scales, either truly were of that opinion or lacked adequate knowledge of the matter [25]. Krosnick et al. (2002) also found that respondents with low levels of education are more likely to choose this type of middle alternative answer option. Furthermore, they suggested that people choose this alternative when they find the task burdensome [26]. This suggests that educational interventions may decrease the tendency for these individuals to perceive their risk as same as others, while their MI risk level remains the same. Awareness of this tendency is important to acknowledge when planning and interpreting studies of risk perception.

This study aimed to identify predictors of cardiovascular risk perception and was built on cross-sectional data. It is therefore not possible to draw conclusions about causal relationships. In prediction models, confounding is not an issue, however, it is crucial that the sample is representative for the population for which the model will used [27]. The age range was rather narrow (50–64). Therefore, the results of this study may not be generalizable to other age groups, especially in younger populations where established CVD is less common and the ten-year risk of a CVD event is in fact very low. Information about CVD risk factors were mainly self-reported, which could induce bias. However, in the case of risk perception, it seems appropriate to focus on risk factors that the individuals believe they have. Unknown risk factors seem unlikely to influence risk perception. The study consisted of a random sample of the general population, which is a strength of the study.

## 5 Clinical implications

Health professionals working with risk calculators should be aware of the variety in interpretation of risk numbers in terms being low or high. They should help the patient with the interpretation by providing a common reference point by comparing the risk with similar peers of same age and sex. Furthermore, it appears that lifestyle and psychosocial factors are not enough for a person to perceive their MI risk as higher than that of others, which should be considered in primary preventive interventions and health communication to the healthy population. Finally, health professionals should underline to patients that they can have an increased MI-risk even when they feel fine, and that the development of CVDs often is silent.

## 6 Conclusions

General health was a common predictor of self-perceived MI risk for all outcomes. Lifestyle factors (BMI, physical activity), and stress dominated the predictors for perceiving MI risk as lower than others, while high cholesterol predicted higher MI risk perception.

## Source of funding

This work was funded by a grant from the 10.13039/100018870Swedish Heart and Lung Association (grant number: 20150049).

## Declaration of competing interest

None.

## References

[1] Lozano R., Naghavi M., Foreman K., Lim S., Shibuya K., Aboyans V., Abraham J., Adair T., Aggarwal R., Ahn S.Y., Alvarado M., Anderson H.R., Anderson L.M., Andrews K.G., Atkinson C., Baddour L.M., Barker-Collo S., Bartels D.H., Bell M.L., Benjamin E.J., Bennett D., Bhalla K., Bikbov B., Bin Abdulhak A., Birbeck G., Blyth F., Bolliger I., Boufous S., Bucello C., Burch M., Burney P., Carapetis J., Chen H., Chou D., Chugh S.S., Coffeng L.E., Colan S.D., Colquhoun S., Colson K.E., Condon J., Connor M.D., Cooper L.T., Corriere M., Cortinovis M., de Vaccaro K.C., Couser W., Cowie B.C., Criqui M.H., Cross M., Dabhadkar K.C., Dahodwala N., De Leo D., Degenhardt L., Delossantos A., Denenberg J., Des Jarlais D.C., Dharmaratne S.D., Dorsey E.R., Driscoll T., Duber H., Ebel B., Erwin P.J., Espindola P., Ezzati M., Feigin V., Flaxman A.D., Forouzanfar M.H., Fowkes F.G., Franklin R., Fransen M., Freeman M.K., Gabriel S.E., Gakidou E., Gaspari F., Gillum R.F., Gonzalez-Medina D., Halasa Y.A., Haring D., Harrison J.E., Havmoeller R., Hay R.J., Hoen B., Hotez P.J., Hoy D., Jacobsen K.H., James S.L., Jasrasaria R., Jayaraman S., Johns N., Karthikeyan G., Kassebaum N., Keren A., Khoo J.P., Knowlton L.M., Kobusingye O., Koranteng A., Krishnamurthi R., Lipnick M., Lipshultz S.E., Ohno S.L., Mabweijano J., MacIntyre M.F., Mallinger L., March L., Marks G.B., Marks R., Matsumori A., Matzopoulos R., Mayosi B.M., McAnulty J.H., McDermott M.M., McGrath J., Mensah G.A., Merriman T.R., Michaud C., Miller M., Miller T.R., Mock C., Mocumbi A.O., Mokdad A.A., Moran A., Mulholland K., Nair M.N., Naldi L., Narayan K.M., Nasseri K., Norman P., O'Donnell M., Omer S.B., Ortblad K., Osborne R., Ozgediz D., Pahari B., Pandian J.D., Rivero A.P., Padilla R.P., Perez-Ruiz F., Perico N., Phillips D., Pierce K., Pope C.A., Porrini E., Pourmalek F., Raju M., Ranganathan D., Rehm J.T., Rein D.B., Remuzzi G., Rivara F.P., Roberts T., De Leon F.R., Rosenfeld L.C., Rushton L., Sacco R.L., Salomon J.A., Sampson U., Sanman E., Schwebel D.C., Segui-Gomez M., Shepard D.S., Singh D., Singleton J., Sliwa K., Smith E., Steer A., Taylor J.A., Thomas B., Tleyjeh I.M., Towbin J.A., Truelsen T., Undurraga E.A., Venketasubramanian N., Vijayakumar L., Vos T., Wagner G.R., Wang M., Wang W., Watt K., Weinstock M.A., Weintraub R., Wilkinson J.D., Woolf A.D., Wulf S., Yeh P.H., Yip P., Zabetian A., Zheng Z.J., Lopez A.D., Murray C.J., AlMazroa M.A., Memish Z.A. (2012). Global and regional mortality from 235 causes of death for 20 age groups in 1990 and 2010: a systematic analysis for the Global Burden of Disease Study 2010. Lancet.

[2] Yusuf S., Hawken S., Ounpuu S., Dans T., Avezum A., Lanas F., McQueen M., Budaj A., Pais P., Varigos J., Lisheng L. (2004). Effect of potentially modifiable risk factors associated with myocardial infarction in 52 countries (the INTERHEART study): case-control study. Lancet.

[3] Piepoli M.F., Hoes A.W., Agewall S., Albus C., Brotons C., Catapano A.L., Cooney M.T., Corra U., Cosyns B., Deaton C., Graham I., Hall M.S., Hobbs F.D., Lochen M.L., Lollgen H., Marques-Vidal P., Perk J., Prescott E., Redon J., Richter D.J., Sattar N., Smulders Y., Tiberi M., van der Worp H.B., van Dis I., Verschuren W.M., De Backer G., Roffi M., Aboyans V., Bachl N., Bueno H., Carerj S., Cho L., Cox J., De Sutter J., Egidi G., Fisher M., Fitzsimons D., Franco O.H., Guenoun M., Jennings C., Jug B., Kirchhof P., Kotseva K., Lip G.Y., Mach F., Mancia G., Bermudo F.M., Mezzani A., Niessner A., Ponikowski P., Rauch B., Ryden L., Stauder A., Turc G., Wiklund O., Windecker S., Zamorano J.L. (2016). European guidelines on cardiovascular disease prevention in clinical practice: the sixth joint task force of the European society of cardiology and other societies on cardiovascular disease prevention in clinical practice (constituted by representatives of 10 societies and by invited experts): developed with the special contribution of the European association for cardiovascular prevention & rehabilitation (EACPR). Eur J Prev Cardiol.

[4] Carpenter C.J. (2010). A meta-analysis of the effectiveness of health belief model variables in predicting behavior. Health Commun..

[5] Usher-Smith J.A., Silarova B., Schuit E., Moons K.G., Griffin S.J. (2015). Impact of provision of cardiovascular disease risk estimates to healthcare professionals and patients: a systematic review. BMJ Open.

[6] Thakkar J., Heeley E.L., Chalmers J., Chow C.K. (2016). Inaccurate risk perceptions contribute to treatment gaps in secondary prevention of cardiovascular disease. Intern. Med. J..

[7] Winkleby M.A., Flora J.A., Kraemer H.C. (1994). A community-based heart disease intervention: predictors of change. Am. J. Publ. Health.

[8] Katz M., Laurinavicius A.G., Franco F.G., Conceicao R.D., Carvalho J.A., Pesaro A.E., Wajngarten M., Santos R.D. (2015). Calculated and perceived cardiovascular risk in asymptomatic subjects submitted to a routine medical evaluation: the perception gap. Eur J Prev Cardiol.

[9] Petr E.J., Ayers C.R., Pandey A., de Lemos J.A., Powell-Wiley T.M., Khera A., Lloyd-Jones D.M., Berry J.D. (2014). Perceived lifetime risk for cardiovascular disease (from the Dallas Heart Study). Am. J. Cardiol..

[10] Claassen L., Henneman L., Nijpels G., Dekker J., Marteau T., Timmermans D. (2007). Causal beliefs and perceptions of risk for diabetes and cardiovascular disease, The Netherlands. Prev. Chronic Dis..

[11] Waldron C.A., van der Weijden T., Ludt S., Gallacher J., Elwyn G. (2011). What are effective strategies to communicate cardiovascular risk information to patients? A systematic review. Patient Educ. Counsel..

[12] Lipkus I.M. (2007). Numeric, verbal, and visual formats of conveying health risks: suggested best practices and future recommendations. Med. Decis. Making.

[13] Frijling B.D., Lobo C.M., Keus I.M., Jenks K.M., Akkermans R.P., Hulscher M.E., Prins A., van der Wouden J.C., Grol R.P. (2004). Perceptions of cardiovascular risk among patients with hypertension or diabetes. Patient Educ. Counsel..

[14] Yamagishi K. (1997). When a 12.86% mortality is more dangerous than 24.14%: implications for risk communication. Appl. Cognit. Psychol..

[15] Edwards A., Elwyn G., Mulley A. (2002). Explaining risks: turning numerical data into meaningful pictures. Bmj.

[16] Grauman Å., Veldwijk J., James S., Hansson M., Byberg L. (2021).

[17] Bergstrom G., Berglund G., Blomberg A., Brandberg J., Engstrom G., Engvall J., Eriksson M., de Faire U., Flinck A., Hansson M.G., Hedblad B., Hjelmgren O., Janson C., Jernberg T., Johnsson A., Johansson L., Lind L., Lofdahl C.G., Melander O., Ostgren C.J., Persson A., Persson M., Sandstrom A., Schmidt C., Soderberg S., Sundstrom J., Toren K., Waldenstrom A., Wedel H., Vikgren J., Fagerberg B., Rosengren A. (2015). The Swedish CArdioPulmonary BioImage Study: objectives and design. J. Intern. Med..

[18] Wangdahl J.M., Martensson L.I. (2014). The communicative and critical health literacy scale--Swedish version. Scand. J. Publ. Health.

[19] McNaughton C.D., Cavanaugh K.L., Kripalani S., Rothman R.L., Wallston K.A. (2015). Validation of a Short, 3-item version of the subjective numeracy scale. Med. Decis. Making.

[20] Grauman Å., Hansson M., James S., Veldwijk J., Höglund A. (2019). Exploring research participants' perceptions of cardiovascular risk information-Room for improvement and empowerment. Patient Educ. Counsel..

[21] Broadbent E., Petrie K.J., Ellis C.J., Anderson J., Gamble G., Anderson D., Benjamin W. (2006). Patients with acute myocardial infarction have an inaccurate understanding of their risk of a future cardiac event. Intern. Med. J..

[22] Astin F., Closs S.J., McLenachan J., Hunter S., Priestley C. (2009). Primary angioplasty for heart attack: mismatch between expectations and reality?. J. Adv. Nurs..

[23] Slovic P., Finucane M.L., Peters E., MacGregor D.G. (2004). Risk as analysis and risk as feelings: some thoughts about affect, reason, risk, and rationality. Risk Anal..

[24] Xu H., Farmer H.R., Granger B.B., Thomas K.L., Peterson E.D., Dupre M.E. (2021). Perceived versus actual risks of 30-day readmission in patients with cardiovascular disease. Circ. Cardiovasc. Qual. Outcomes.

[25] Sturgis P., Roberts C., Smith P. (2014). Middle alternatives revisited: how the neither/nor response acts as a way of saying “I don't know”. Socio. Methods Res..

[26] Krosnick J.A., Holbrook A.L., Berent M.K., Carson R.T., Hanemann W.M., Kopp R.J., Mitchell R.C., Presser S., Ruud P.A., Smith V.K., Moody W.R., Green M.C., Conaway M. (2002). The impact of "No opinion" response options on data quality: non-attitude reduction or an invitation to satisfice?. Publ. Opin. Q..

[27] Schooling C.M., Jones H.E. (2018). Clarifying questions about "risk factors": predictors versus explanation. Emerg. Themes Epidemiol..

